# Supplementary material for: Conceptualizing Vulnerability for Health Effects of the COVID-19 Pandemic and the Associated Measures in Utrecht and Zeist: A Concept Map
Source: Int J Environ Res Public Health. 2021 Nov 19;18(22):12163. doi: 10.3390/ijerph182212163 (PMC8621190; doi:10.3390/ijerph182212163)
Supplement: Supplementary file 1 [file ijerph-18-12163-s001.zip › ijerph-1451465-supplementary.pdf]

Supplementary Table S1. Statements in each cluster and the average ranking of the statements.

No.	Cluster label	No.	Statement	Ranking
1	Activities	41	That the possibility to exercise and move is lost	3.50
		59	That nice things fall away	3.38
		1	Not being able to do volunteer work anymore	3.06
		10	Not having a daily routine	3.56
		93	The closing of schools	3.63
		95	Stagnation of your development	3.25
		29	Avoiding school	3.69
		9	Losing your daily routine	3.69
		11	The disappearance of external incentives to do something	3.44
		64	Boredom	2.73
2	Risk groups	26	Fear of getting corona	3.38
		24	Being homeless	3.81
		92	Getting a short fuse because of the measures and fear	3.19
		51	Being a youth	3.50
		81	Being an informal caregiver	3.31
		44	Being a senior citizen	4.13
		80	Being a pupil or student	3.25
3	Social environment	97	That loneliness increases	4.19
		6	That social isolation among people is increasing	4.31
		21	Having a good relationship with a person with reduced immunity	3.00
		61	Lack of physical contact (intimacy/'skin hunger')	3.56
		72	Not being able to build a social network	4.00
		33	Living in an institution	3.13
		87	Not being happy in your home situation	3.44
		28	Not being able to go to an association or club anymore	3.63
		66	Changing your living environment during the corona pandemic	2.06
4	Personal environment	77	Living on your own	3.69
		83	Being removed from home as a child	3.31
		8	Not having close or intimate ties with one or more people	4.13
		36	The lack of a supportive social environment	4.56
		37	The lack of a suitable physical environment	3.44
		34	The lack of informal help	3.88
		30	Avoiding contacts	3.73
		12	Being in an unsafe (home) situation	4.81
		47	Not being happy in your relationship	2.87
5	Finances	15	A low income	3.13
		76	Having financial worries	4.31
		16	Having debts	3.60
		23	Not having the right to social benefits in the Netherlands	3.50
		2	Not having the financial means to follow the measures	3.94
6	Work and income	55	Fear of losing your job or work	3.13
		56	Fear of losing your income	3.63
		13	A flexible contract	3.13
		45	Losing income due to corona	4.50
		88	Losing your job	4.56
		74	Being a small entrepreneur in sectors affected by the corona measures	4.06
		19	Prolonged working under (extra) pressure	3.80
		25	Being completely dependent on your employer for your basic services (such as housing)	3.50
		14	Being self-employed	2.81
7	Perception of work	86	Working as a teacher	2.63
		31	Having a contact profession	3.19
		73	Having a young family and a job	2.69
		48	Being a workaholic	2.00
		84	Working in a sector that has been affected by the corona measures	3.94
		20	Long-term working from home	2.88
		57	Difficulty finding a job	3.63
		98	Starting a new job during the corona pandemic	1.69
		94	Not being happy in your work situation	2.50
8	Knowledge, skills and attitude	18	Being influenced by cultural norms and values that conflict with the corona measures	3.38
		17	Being influenced by conflicting messages about corona and the measures	3.13
		5	That people think corona doesn't exist	2.81
		32	Consulting a limited number of information sources	3.19
		4	Not trusting government agencies	2.94
		38	Lack of digital skills or resources	3.27
		3	Difficulty understanding the measures (due to language barrier)	3.94
		63	Not really understanding what's happening	3.67
		67	Not knowing anything about the current situation/pandemic	3.63
		91	Incomprehension of the measures	2.63
9	Mental health	82	Having a mild intellectual disability	3.60
		42	An unhealthy lifestyle	3.25
		89	Sensitivity to the feelings of others	2.29
		50	Having psychological complaints or disorders	4.25
		39	Not seeing a personal perspective	3.56
		43	The lack of discipline for a healthy lifestyle	3.13
		65	Not being able to or not daring to express your identity	2.44
		60	Having differences of opinion in your immediate environment about corona	3.19
		70	Difficulty adapting to new situations	3.50
		49	Psychological frailty	4.56
		75	Being suicidal	4.38
		79	Having addiction problems	3.75
10	Personal consequences	46	Being dependent on public transport	2.50
		7	That people are less able to deal with stress	3.44
		58	An accumulation of problems	4.19
		68	Losing health due to corona	4.06
		69	The (partial) cessation of campaigns for charities that support vulnerable groups	2.94
		71	Traumatic experiences due to the measures	3.56
11	Physical health	78	Being (severely) overweight	3.19
		53	Being unable to wear a mask for psychological or medical reasons	2.69
		90	Having burnout complaints	3.50
		52	Having physical ailments or limitations	3.81
		85	Having a lot of stress	3.94
		22	Having an increased risk of contracting corona yourself	4.06
12	Consequences for health and care	40	That healthcare is less available	4.25
		27	Avoiding or delaying care	4.31
		35	The lack of professional help	4.19
		54	The loss of loved ones due to corona	4.25
		62	Dealing with long-term corona complaints	4.19
		96	Traumatic experiences due to the disease COVID	4.06
